# Apocrine Hidrocystoma of the Breast Mimicking an Epidermal Inclusion Cyst

**DOI:** 10.7759/cureus.114732

**Published:** 2026-08-18

**Authors:** Sarah E Rashdan, Derek Brook, Yve Huttenbach, Ida Orengo

**Affiliations:** 1 Dermatology, Baylor College of Medicine, Houston, USA; 2 Radiology, Baylor College of Medicine, Houston, USA; 3 Pathology, Baylor College of Medicine, Houston, USA

**Keywords:** apocrine hidrocystoma, breast, cutaneous adnexal tumor, dermatopathology, diagnostic pitfall, epidermal inclusion cyst

## Abstract

Apocrine hidrocystomas are rare, benign cystic tumors arising from apocrine sweat glands, with a predilection for the head and neck, most commonly involving the eyelids. Occurrence within breast tissue is exceptionally uncommon and represents a diagnostic challenge, particularly given the high prevalence of other cystic lesions in this location. We report a 67-year-old female who presented for excision of a new, painless right breast nodule detected on self-examination. She had a known history of dense breast tissue and benign breast masses. Ultrasonography identified a hypoechoic lesion consistent with an epidermal inclusion cyst. Clinical examination revealed a firm, flesh-colored nodule with a small light-brown macule on the overlying skin, further supporting this diagnosis. During excision, clear watery fluid was expressed, an atypical intraoperative finding inconsistent with the expected thick keratinaceous contents of an epidermal inclusion cyst. Histopathologic analysis demonstrated a multiloculated cyst lined by apocrine epithelium with characteristic features, confirming the diagnosis of apocrine hidrocystoma. This case contributes to the limited literature on breast apocrine hidrocystoma and underscores the value of clinicopathologic correlation when evaluating cystic lesions in atypical anatomic locations.

## Introduction

Apocrine hidrocystomas are rare, idiopathic benign cystic tumors of apocrine sweat glands with a predilection for the head and neck, most commonly involving the eyelids [1]. Clinically, they present as dome‑shaped lesions with a bluish hue and are usually solitary. Histologically, these tumors demonstrate unilocular or multilocular cystic spaces lined by a bilayered epithelium with characteristic apical decapitation (“snouting”) [2]. Less commonly, apocrine hidrocystomas have been reported in sites, such as the axilla, groin, and scalp. Involvement of the breast is particularly uncommon, with only isolated cases described in the literature. By contrast, epidermal inclusion cysts are relatively common cutaneous lesions of the breast and may share overlapping clinical and imaging features with apocrine hidrocystomas. Here, we present a case of apocrine hidrocystoma of the breast clinically mimicking an epidermal inclusion cyst, highlighting a diagnostic pitfall in the evaluation of cystic breast lesions.

## Case presentation

A 67-year-old female presented to the dermatology clinic for excision of a right breast nodule, which she detected during a routine self-breast exam. She described a new, painless mass located near a previously known cyst. The patient had a history of dense breast tissue and benign breast masses identified on prior mammography. Breast ultrasonography revealed a 4 × 3 × 4 mm hypoechoic lesion within the dermis connected to a deeper 8 × 4 × 6 mm hypoechoic component, consistent with an epidermal inclusion cyst at the 4 o’clock position (Figure 2). Due to perceived interval growth, the patient proceeded with surgical excision.

**Figure 1:**
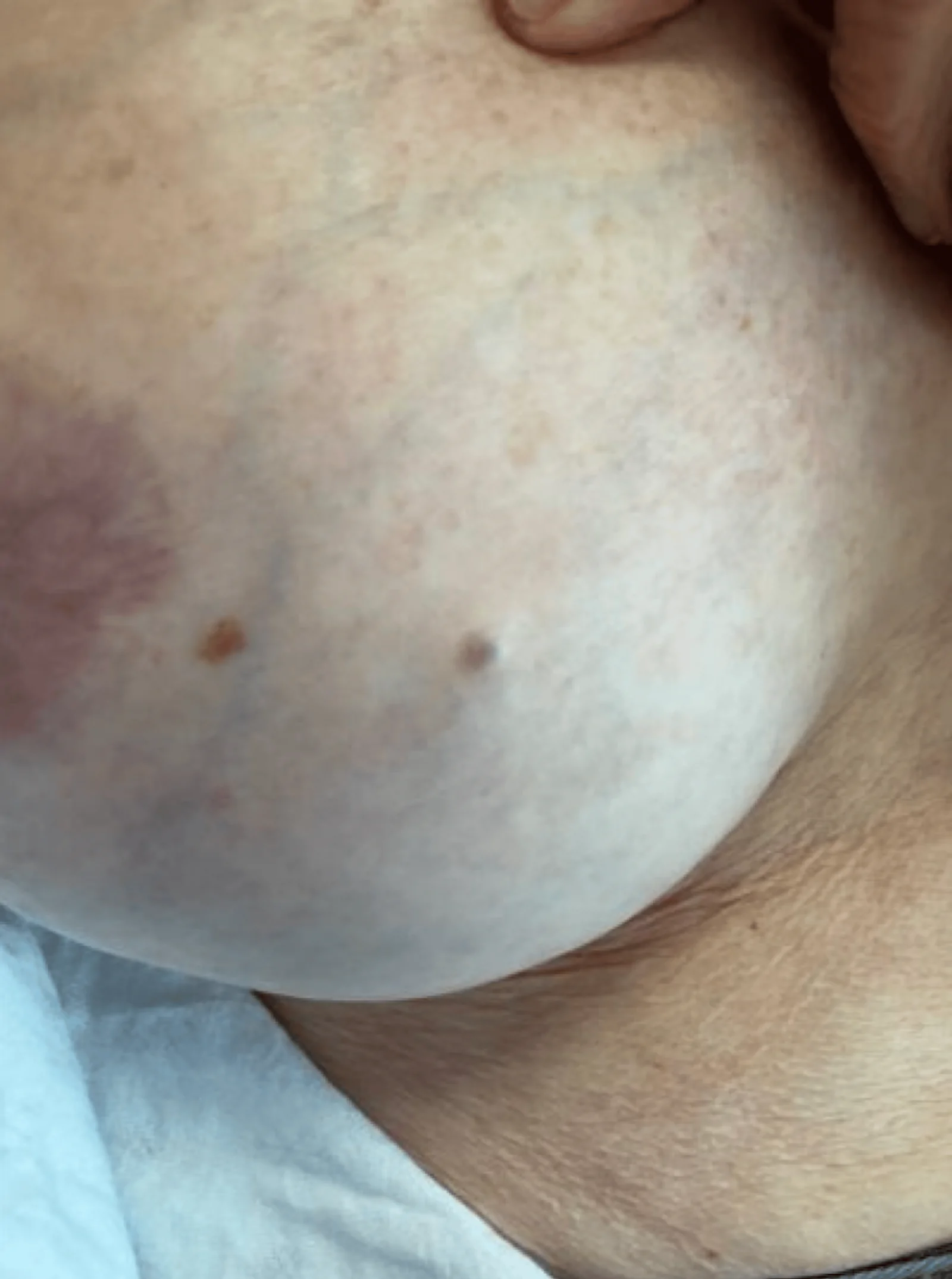
Clinical appearance of a firm, flesh-colored nodule on the right breast with subtle overlying pigmentation.

**Figure 2:**
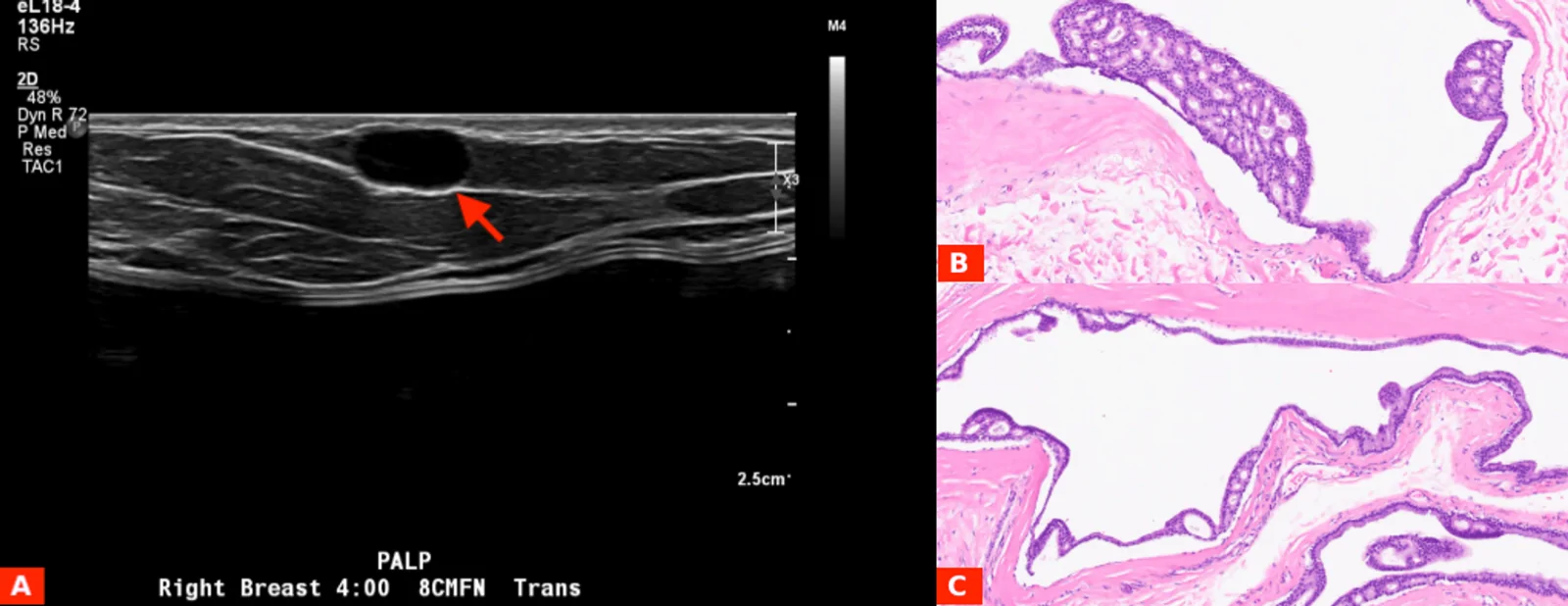
(A) Breast ultrasonography demonstrating a hypoechoic dermal lesion with a deeper component. (B,C) Histopathologic sections demonstrating a multiloculated cystic lesion lined by apocrine cells, consistent with apocrine hidrocystoma (H&E stain).

Examination revealed a firm, flesh‑colored nodule with a small, light‑brown macule on the overlying skin (Figure 1). During excision, the lesion was transected and expressed clear, watery fluid. This finding is unusual for an epidermal inclusion cyst, in which thick, keratinaceous material would be expected. The specimen was submitted for histopathologic evaluation. Microscopic examination demonstrated a multiloculated cystic structure lined by apocrine cells, without features suggestive of malignancy, findings consistent with apocrine hidrocystoma (Figure 2).

## Discussion

Apocrine hidrocystomas are benign cystic tumors of apocrine sweat gland origin that may clinically resemble a variety of common cutaneous lesions, complicating diagnosis when in atypical locations. In this case, diagnosis was complicated by the lesion’s location within the breast, an area with a high incidence of epidermal inclusion cysts. The lesion’s firm consistency, absence of translucent or bluish discoloration, and the presence of a small light‑brown macule on the overlying skin favored a clinical diagnosis of epidermal inclusion cyst. In addition, the overlap in imaging characteristics between these entities limited the diagnostic utility of ultrasonography. Definitive distinction between apocrine hidrocystoma and epidermal inclusion cyst requires histopathologic evaluation, as clinical and radiographic findings may overlap.

Apocrine hidrocystomas involving the breast are rare, with only a small number of cases in the literature to date. Previously described lesions include a large pediatric apocrine hidrocystoma of the breast parenchyma measuring 7 × 6 cm [3] and an adult breast lesion presenting as a 17‑mm papule with associated red‑violet overlying skin changes [4]. A few cases of apocrine hidrocystoma affecting the nipple have been reported, representing a distinct subset in apocrine‑rich tissue of the nipple-areolar complex, separate from lesions arising within the breast itself [5].

Unlike previously reported cases, the present case involved breast tissue rather than the nipple, was of typical size for an apocrine hidrocystoma, and lacked overlying skin changes. The lesion was clinically and radiographically interpreted as an epidermal inclusion cyst, highlighting a distinct diagnostic pitfall in the evaluation of cystic breast lesions. To our knowledge, this case represents one of the few reported instances of apocrine hidrocystoma in breast tissue mimicking an epidermal inclusion cyst.

## Conclusions

This case emphasizes the uncommon presentation of apocrine hidrocystoma involving the breast and highlights its potential to mimic other common cystic lesions, including epidermal inclusion cysts. Although benign, overlapping clinical and imaging features reinforce the importance of histopathologic evaluation, particularly when intraoperative findings are atypical. This case contributes to the limited literature on breast apocrine hidrocystoma and demonstrates the value of clinicopathologic correlation in accurately diagnosing breast lesions.
